# Association between single nucleotide polymorphisms of DNA repair genes (BRCA1, BRCA2, and PALB2) and breast cancer incidence in a subset of Iranian population

**DOI:** 10.1186/s13053-025-00311-0

**Published:** 2025-03-29

**Authors:** Sepideh Jahangiri, Zahra Abdan, Massoud Houshmand, Ali Souroush, Mozaffar Aznab

**Affiliations:** 1 Clinical Research Development Center of Imam Reza Hospital, Kermanshah University of Medical Sciences, Kermanshah, Iran; 2 Department of Medical Genetics, National Institute of Genetics and Biotechnology, Tehran, Iran; 3 Department of Medical Physics, Kermanshah University of Medical Sciences, Kermanshah, Iran; 4 Medical Oncology- Hematology, Internal Medicine Department, Kermanshah University of Medical Sciences, Kermanshah, Iran

**Keywords:** Breast cancer, BRCA1, BRCA2, PALB2, Polymorphism

## Abstract

**Background:**

Breast cancer (BC) is the most common malignancy among Iranian females, accounting for 24.4% of all malignancies. Germ line mutations in DNA repair system-related genes are associated with an increased risk of BC. This study aims to evaluate the frequencies of single nucleotide polymorphisms (SNPs) in the BRCA1, BRCA2, and PALB2 genes in patients with BC from a subset of the Iranian population in the western part of Iran.

**Methods:**

Blood samples were collected from 335 patients with BC and 354 healthy matched volunteers. Genomic DNA was extracted using the salting-out method and, after quality control, was genotyped using the multiplex TaqMan allelic discrimination assay for three SNPs: rs80359550 (6174 delT) in the BRCA2 gene, rs180177102 in the PALB2 gene, and rs386833395 (185delAG) in the BRCA1 gene. Statistical analysis was performed to examine allele frequency, odds ratio, and relative risk (genetic association) in a retrospective case-control study.

**Results:**

The data showed no association between rs386833395 and BC risk in the studied population (odds ratio = 1), whereas rs80359550 and rs180177102 polymorphisms were strongly associated with BC risk in patients (odds ratio = 0.01 for both, with *p*-values of 0.011 and 0.021, respectively).

**Conclusions:**

Our findings suggest no significant association between the rs386833395 polymorphism and BC risk in the Iranian Kurdish population, while rs80359550 and rs180177102 polymorphisms were strongly associated with BC. However, the study has several limitations, including its retrospective design, a relatively small sample size, and the potential lack of generalizability to other ethnic groups within Iran. Future studies involving larger cohorts and more diverse populations are needed to confirm these results.

## Introduction

Breast cancer (BC) is the most commonly diagnosed cancer among females and remains the leading cause of cancer-related deaths worldwide [1]. In 2018, over 2 million new BC cases were recorded globally, with projections estimating this number will reach 3.2 million by 2050 [2, 3]. In Iran, BC is also the most prevalent cancer among females, accounting for 24.4% of all cancer cases. Its incidence rate varies significantly by race, ethnicity, and geographic region [4]. BC is a heterogeneous disease influenced by a range of factors, including age, environmental exposures, hormonal status, lifestyle, family history, and genetic factors [4]. Early identification of genetic markers associated with BC susceptibility is a critical area of cancer genetics research [5]. Genetic polymorphisms have been identified as significant contributors to BC susceptibility, and various studies have investigated the association of single nucleotide polymorphisms (SNPs) in genes involved in BC development. For example, research on the HOTAIR gene has shown that specific polymorphisms, such as rs920778 and rs12826786, are significantly linked to breast cancer susceptibility and clinicopathological features in Turkish populations [6, 7]. Additionally, SNPs in other genes, like hTERT, have been explored in relation to BC risk, further enhancing our understanding of the genetic basis of BC susceptibility [8]. These findings underscore the importance of studying genetic variations in diverse populations to better understand their role in BC risk.

Germ line mutations account for 5–10% of all BC cases and approximately 30% of familial BC cases in well-known BC susceptibility genes, such as BRCA1 and BRCA2, with frequency varying by ethnicity [9]. These genes are critical in the repair of double-strand breaks (DSB) through the non-homologous end-joining (NHEJ) and homologous recombination (HR) DNA repair pathways [10]. Proteins involved in the HR pathway, such as Ataxia telangiectasia mutated (ATM), ATR, and BRCA1/2, play essential roles in detecting and repairing DNA damage [11]. HR pathway involves proteins that can diagnose broken ends such as Ataxia telangiectasia mutated (ATM), ataxia-telangiectasia, and Rad3 related (ATR) and repair DNA damages such as BRCA2 and BRCA1 [11, 12]. PALB2, a key mediator in the HR pathway, forms protein-protein interactions with BRCA2 and other repair proteins to facilitate DNA repair [9, 13]. Polymorphisms in these genes, which affect their expression and/or function, can influence BC risk [4]. Previous studies have shown that SNPs in BRCA1, BRCA2, and PALB2 genes are associated with BC risk [14]. The frequency of these SNPs varies across different populations, depending on ethnic background, highlighting the need to investigate their prevalence in diverse populations [15–17]. To date, there is limited data on the mutation frequencies of BRCA1, BRCA2, and PALB2 in BC patients from the western part of Iran. This study aims to investigate the frequencies of the BRCA2 (rs80359550), PALB2 (rs180177102), and BRCA1 (rs386833395) SNPs in both BC patients and healthy individuals from the Kurdish population in Kermanshah province, Iran.

## Materials and methods

### Population study

In this study, 335 women with confirmed breast cancer, aged 23 to 79 years, were included. All patients were enrolled in 2017, with 218 patients from Imam Reza Hospital in Kermanshah, Iran, whose diagnoses were based on standard clinical and histopathological data reported in their medical records. The study also randomly included 354 age-matched, cancer-free controls without a family history of cancer or underlying disorders, who visited the same hospital for regular physical examinations.

The sample size of 335 breast cancer patients and 354 healthy controls was determined based on previous studies on genetic associations with breast cancer. Given the expected minor allele frequencies of the SNPs under investigation and a moderate effect size (OR = 1.5), this sample size was deemed sufficient to detect statistically significant associations with a reasonable degree of power. Demographic and clinical data, including age, family history of breast cancer, tumor histology, tumor stage and grade, number of children, estrogen receptor (ER), progesterone receptor (PR), HER2, and P53 status, were obtained for each participant from archived medical records. All patients and control participants were residents of Kermanshah and of Kurdish ethnicity. Ethical approval for the study was obtained from the Ethics Committee of Kermanshah University of Medical Sciences, and informed consent was provided by all participants.

### DNA extraction

Five milliliters of peripheral blood samples were obtained from both the breast cancer patients and control individuals, collected in EDTA tubes. Genomic DNA was then isolated from the blood nucleated cells using a salting-out method according to standard protocols [18]. All extracted DNA samples were stored at − 20 °C until they were used for genotyping analysis.

### SNP genotyping

Genotyping of three SNPs, rs386833395 (BRCA1 gene), rs80359550 (BRCA2 gene), and rs180177102 (PALB2 gene), was performed using the multiplex TaqMan allelic discrimination assay (Applied Biosystems, USA) with SDS version 2.3 software. Polymerase chain reaction (PCR) reactions were conducted in a total volume of 25 µL, containing specific TaqMan^®^ Genotyping Assays (Applied Biosystems, USA), TaqMan^®^ Universal PCR Master Mix, and 1–10 ng of genomic DNA. The thermal cycling conditions for amplification included an initial denaturation step at 92 °C for 10 min, followed by 45 cycles of 30 s at 92 °C, 45 s at 60 °C, and 1 min at 72 °C.

### Statistical analysis

The odds ratio (OR) with 95% confidence intervals (95% CI) was used to assess the association between BRCA1, BRCA2, and PALB2 polymorphisms and the risk of breast cancer (BC) in both patients and healthy controls, as calculated according to the Altman guide [19]. The Chi-square goodness-of-fit test was employed to evaluate deviations in allelic frequencies from Hardy-Weinberg equilibrium (HWE), expressed as p² + 2pq + q² = 1. An odds ratio close to 1 indicates no significant risk associated with the disease. Multiple inheritance models (including co-dominant, dominant, recessive, over dominant, and log-additive models) were applied to assess the significance of each genotype. Statistical analysis was conducted using GraphPad Prism version 8.0.2 for Windows (GraphPad Software, San Diego, California, USA; www.graphpad.com), and a two-sided *p*-value of less than 0.05 was considered statistically significant (Table 1).

**Table 1:** The primer and probe sequences

	rs80359550 (B)	rs180177102 (A)	rs386833395
Forward primer	ACGATTCACATAAGGTTTTTGC	CTAAAGTCAGCTCTCCCGCT	GGACGTTGTCATTAGTTCTTTGGT
Reverse primer	TGTCTTGCGTTTTGTAATGAAGCA	CGTGCTGATATTTGTGTGAGGT	TTCCCGGACCACAGGATTTG
Probe 1	AGCACAGCAAGTGGAAAATCTGTCCA	ATCATTGTGAACCACTTTTGCCAAC	GCAGAAAATCTTAGAGTGTCCCATC
Probe2	AGCACAGCAAGGGAAAATCTGTCCA	ATCATTGTGAACCACTTTGCCAAC	GCAGAAAATCTTTGTCCCATC

## Results

### Demographic and clinical profile

A total of 335 breast cancer (BC) patients and 354 healthy controls from an Iranian population with similar ethnic backgrounds were included in this study. The mean age of BC patients was 46.6 ± 23.2 years, while the mean age of controls was 52.2 ± 10.4 years. The demographic and pathological characteristics of the BC patients, including tumor histology, stage, and tumor behavior, are summarized in Table 2. Approximately 54% of the BC patients had a family history of cancer, and 97.6% had a ductal histological subtype. Of the patients, 228 (68%) were in stages I-II, while 107 (31.9%) were in stages III-IV (Table 2).

**Table 2:** Demographic and pathologic characteristics of breast cancer patients

	Controls (*n*=354) n(%)	Patients (*n*=375) n (%)
**Age of onset **(Mean±S.D)	52.2 ± 10.4	49.6 ±13.2
**Race**	Kurdish	Kurdish
**Family history**	-	
**Yes **		204 (54.4%)
**No **		171 (45.6%)
**Tumor histology:**	-	
Ductal		327 (97.6%)
Lobular		8 (2.3%)
**Clinical stage: **	-	
Stages I-II		228 (68%)
Stages III-IV		107 (31.9)
**LN grade:**	-	
0		102 (30.4%)
1		55 (16.4%)
2		59 (17.6%)
>=3		120 (35.8%)
**Number of Children:**	-	
0		4
1		32
2		91
>=3		204
**Metastasis:**	-	
**Yes **		62 (16.5%)
**No **		313 (83.4%)
**Recurrence:**	-	
**Yes **		21 (5.6%)
**No **		354 (94.4%)

### Molecular histopathological markers

The distribution of molecular and histopathological markers in breast cancer patients is presented in Table 3. More than half of the patients were diagnosed as HER2-positive. Additionally, 67.1%, 71.6%, 94%, and 59.1% of the breast cancer patients tested positive for estrogen receptor (ER), progesterone receptor (PR), Ki-67, and p53 status, respectively (Table 3).

**Table 3:** Molecular histopathological markers

	Patients (*n*=375) n (%)
**HER2:**	
Positive	184 (54.9%)
Negative	151 (45%)
**ER status:**	
Positive	225 (67.1)
Negative	110 (32.8)
**PR status:**	
Positive	240 (71.6)
Negative	95 (28.3)
**Ki-67 status:**	
Positive	315 (94.0)
Negative	19 (5.7)
**P53 status:**	
Positive	198 (59.1)
Negative	136 (40.5)

*HER2* Human epidermal growth factor receptor 2, *ER* the estrogen receptor, *PR* progesterone receptor

### SNP associations

**Fig. 1:**
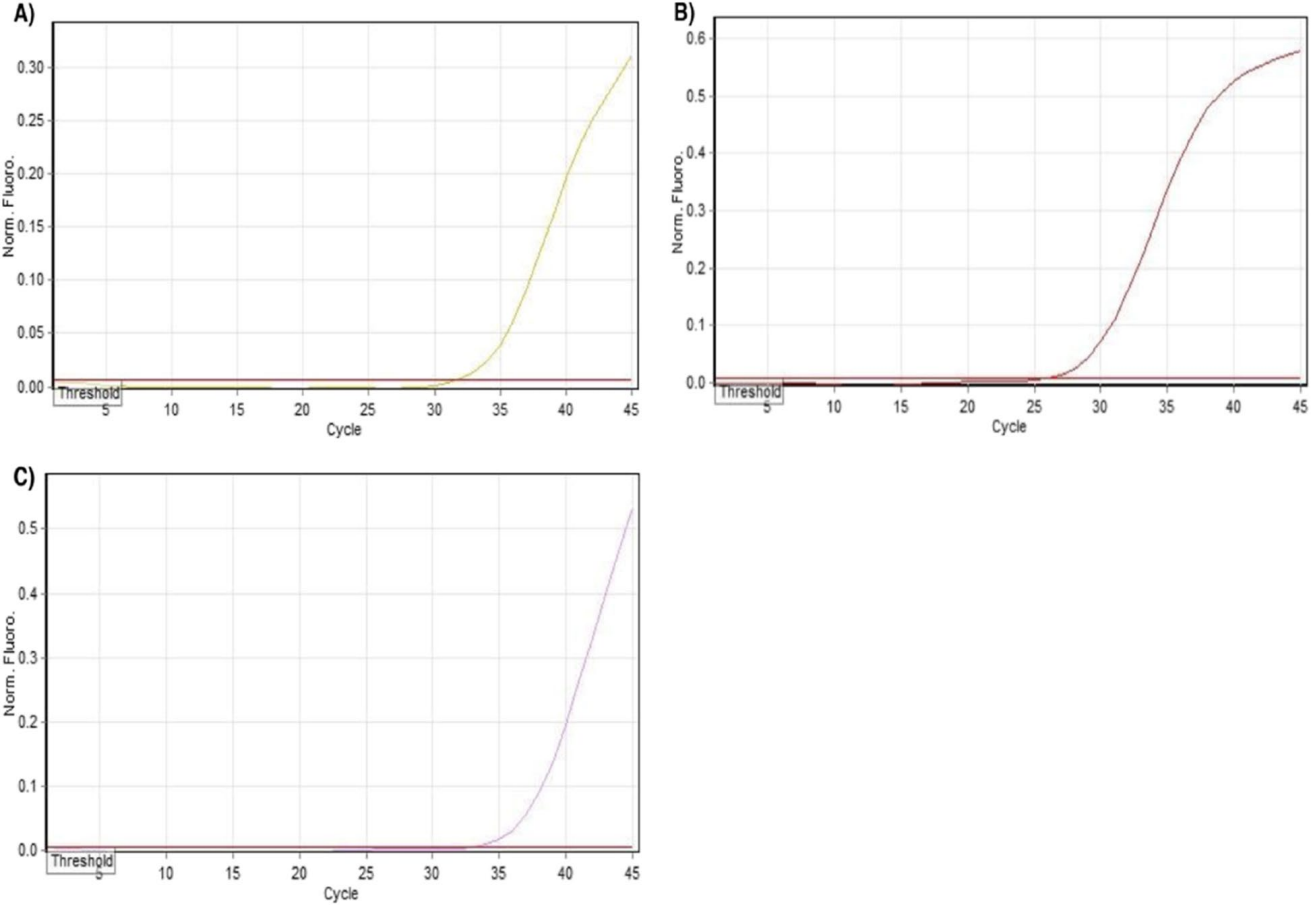
Melt curves derived from Taqman allelic discrimination assay revealed single nucleotide variations in rs180177102 (**A**), rs80359550 (**B**), and rs386833395 (**C**)

Genotypes were detected using a TaqMan^®^ probe-based assay on multiplex PCR (Fig. 1). Three single nucleotide polymorphisms (SNPs)—BRCA1/rs386833395, BRCA2/rs80359550, and PALB2/rs180177102—were genotyped simultaneously in 335 breast cancer (BC) patients and 354 healthy controls, with the distribution of these SNPs shown in Table 4.

**Table 4:** Variant frequencies of rs80359550, rs180177102 and rs386833395 polymorphisms and their association with breast cancer

Allele frequency	Mutant allele frequency	Chi-square value	Odds ratio	95% CI	Relative Risk	Z	*P*-value
rs80359550 (6174delT; BRCA2)	0.004	6.396	0.000	0.000 to 0.659	0.48	2.529	0.011
rs180177102 (c.1592delT; PALB2)	0.002	4.322	0.000	0.000 to 0.630	0.48	2.30	0.021
rs386833395 185delAG (BRCA1)	0	-	1.0 (reference)	0.02 to 53.40	1.0 (reference)	0.028	1.0 (reference)

Our analysis found that the rs386833395 polymorphism in BRCA1 was not detected in either the BC patients or the healthy controls.

For the BRCA2 gene, genotyping of the rs80359550 polymorphism revealed that the mutant genotype (T-) was present in 1% of the patient group, while no mutation was detected in the healthy control group (Table 4). Similarly, for the rs180177102 polymorphism, the mutant allele was found in 0.01 of the BC patients, with no detection in the healthy control group.

To assess the association of these variants with BC risk, we calculated the Odds Ratio (OR) and Relative Risk (RR). No association was observed between rs386833395 and BC risk in either group, as no variation was identified in either the patients or the controls (OR = 1.0; *p*-value = 1.0). However, our results demonstrated that the rs80359550 and rs180177102 polymorphisms were strongly associated with an increased risk of developing breast cancer (OR = 0.000, RR = 0.48 for both; *p*-values = 0.011 and 0.021, respectively) in the studied population (Table 4).

## Discussion

In our study, we observed that the rs80359550 polymorphism in BRCA2 was strongly associated with breast cancer risk in the western population of Iran. These findings align with other studies, including those by Bayram et al. [6], who found significant associations between the HOTAIR rs920778 polymorphism and BC susceptibility in Turkish populations [6]. Similarly, Bayram et al. [7] identified the rs12826786 C > T polymorphism in HOTAIR as a key factor in BC risk and clinicopathological features in another Turkish cohort [7]. These studies further underline the role of genetic polymorphisms in shaping individual susceptibility to BC.

In contrast, we did not detect any association between the rs386833395 polymorphism and BC risk in our study population. This lack of association is similar to the findings of a Turkish study by Aydin et al. [8], which investigated the role of hTERT gene polymorphisms in BC risk. Although this study did not find any significant links between hTERT polymorphisms and BC risk, it supports the notion that genetic associations can vary significantly across different populations [8].

The current study aimed to examine the role of specific polymorphisms in breast cancer risk, focusing on the association between three SNPs—rs4135113, rs4135050, and rs4135066—and BC risk in a very selective subset of the Iranian population. According to our data, the rs80359550 and rs180177102 polymorphisms were strongly associated with BC risk in the western population of Iran. However, no detectable association was found for rs386833395, as the mutation was not observed in either the patient or control groups.

Breast cancer is considered one of the most significant challenges for healthcare systems, with the number of new BC cases increasing worldwide [3]. Late diagnosis is a major contributor to the high mortality rate among these patients [5]. Given the differences among populations based on ethnicity and geographic region, a specific genetic marker panel would facilitate early diagnosis for populations at genetic risk for BC [19] Many studies have reported the importance of genetic polymorphisms in an individual’s susceptibility to BC [20]. Germ line pathogenic variants in DNA repair system genes, such as PALB2, BRCA1, and BRCA2, are correlated with high BC risk [9, 14]. The BRCA1 and BRCA2 genes play a crucial role in double-stranded DNA break repair in response to DNA damage, and germ line mutations in these genes are commonly observed in BC [9, 21, 22]. The prevalence of the 185delAG mutation in BRCA1 and the 6174delT mutation in BRCA2 varies considerably across ethnic populations [9]. Mutation screening in these genes aids in identifying individuals who carry pathogenic variants and are at high risk for developing BC [23].

In our study, we examined the breast cancer susceptibility associated with two SNPs in BRCA1 (rs386833395) and BRCA2 (rs80359550) through a case-control study involving 335 BC patients and 354 healthy controls. We found a stronger association of rs80359550 with breast cancer development compared to previous reports [24]. In contrast, a Turkish study indicated that the rs386833395 polymorphism is not common in the Turkish population with early-onset BC [25]. However, as noted earlier, our results showed a strong association for rs80359550. In another Iranian study, the frequency of the 185delAG (rs386833395) mutation in BRCA1 and the 6174delT (rs80359550) mutation in BRCA2 was examined to assess their impact on BC risk. This study, which included 200 healthy controls, 250 sporadic BC cases, and 55 familial BC cases from southern Iran, found that these variants had a lower frequency in southern Iranian BC patients [26].

Like BRCA1 and BRCA2, the PALB2 gene is one of the ten genes associated with BC risk, with a frequency of 0.7 − 1.1% in familial BC cases [27]. PALB2 encodes a protein that binds and forms a multi-protein complex with BRCA2 and BRCA1, facilitating DNA repair in the homologous recombination pathway [28, 29]. Any germ line variant in the PALB2 gene that leads to an incomplete protein can impair the double-strand break repair system, increasing BC risk [28, 29]. Over the past two decades, population-based studies have indicated that pathogenic variants in PALB2 account for approximately 0.83–2% of familial BC cases in Spanish and African populations [30–32]. Our study provides evidence that the PALB2 polymorphism, rs180177102, is strongly associated with BC risk (OR = 0.012). (OR = 0.012).

This study has several limitations that should be acknowledged. While the sample size is significant, it may still limit the generalizability of the findings, particularly for low-frequency polymorphisms. Additionally, the study focused solely on a subset of the Iranian Kurdish population, which may not reflect the genetic diversity of other populations. The investigation was restricted to a limited number of SNPs and did not include other potential genetic variants or functional analyses to elucidate biological mechanisms. Environmental and lifestyle factors were not considered, and the case-control design may introduce biases. Moreover, breast cancer subtype-specific analyses were not conducted, which could have provided more detailed insights into genetic predispositions for different forms of the disease. Future studies should address these gaps by expanding the sample size, genetic scope, and considering additional factors such as the environment and cancer subtypes.

## Conclusion

The Multiplex Taqman assay for the rs80359550 (BRCA2) and rs180177102 (PALB2) variants has shown a strong correlation with breast cancer risk. However, no association was observed between the rs386833395 variant of BRCA1 and BC susceptibility in the studied Iranian subpopulation. Sensitivity and specificity analysis revealed that both the rs80359550 and rs180177102 variants could be highly beneficial for breast cancer diagnosis. High-throughput studies are necessary to discover more effective and/or founder effect polymorphisms in the cohort of interest. It is important to note that no other similar study has been published on the frequency of the studied polymorphisms and BC risk in such a large cohort of the Iranian population to date. Nevertheless, this study leaves room for future research to expand and strengthen epidemiological studies by using larger sample sizes.

## Data Availability

No datasets were generated or analysed during the current study.
